# Surgical Management of Ovarian Masses in Children: A Comparative Analysis by Pediatric Surgeons and Gynecologists at Two Academic Hospitals in Johannesburg

**DOI:** 10.1002/cnr2.70396

**Published:** 2025-11-05

**Authors:** Nkhensani C. Mashaba, Langanani Mbodi, Ellen M. Mapunda, Tanvier Omar, Derek S. Harrison

**Affiliations:** ^1^ Department of Pediatric Surgery, School of Clinical Medicine, Faculty of Health Sciences University of the Witwatersrand Johannesburg South Africa; ^2^ Department of Pediatric Surgery Polokwane‐Mankweng Tertiary and Academic Hospital, University of Limpopo Polokwane South Africa; ^3^ Department of Gynecologic Oncology, School of Clinical Medicine, Faculty of Health Sciences University of the Witwatersrand Johannesburg South Africa; ^4^ Division of Anatomical Pathology, School of Pathology, Faculty of Health Sciences University of the Witwatersrand Johannesburg South Africa

**Keywords:** laparoscopic surgery, ovarian mass, ovarian preservation, pediatric gynecology, pediatric surgery

## Abstract

**Background and Objectives:**

Existing literature on ovarian masses necessitating intervention in children by pediatric surgeons and gynecologists in Low‐ and Middle‐Income Countries is sparse and lacks collaborative standardization in management between the two subspecialties. Therefore, this study seeks to assess the range of ovarian masses presenting to these two specialties and to explore variations in management.

**Methods:**

A 15‐year retrospective review of surgically biopsied or excised ovarian masses between subspecialties at two academic hospitals in Johannesburg.

**Results:**

We identified 288 patients, six with bilateral disease and 294 ovarian masses. The mean age was 13.34 years (SD ±5.12). The most common presentation was abdominal pain in 149/288 (51.74%); 117 patients (40.62%) were from pediatric surgery and 171 (59.38%) from gynecology departments. There were 127/288 (44.09%) non‐neoplastic and 161/288 (55.90%) neoplastic lesions, of which 110/161 (68.33%) were benign and 51/161 (31.67%) malignant. The neoplastic lesions consisted of 107/161 (66.45%) germ cells, 28/161 (17.39%) surface epithelial tumors, and 26/161 (16.14%) sex cord‐stromal tumors. Ovarian‐sparing surgery was done in 56/288 (19.44%) patients, 22/117 (18.80%) in pediatric surgery, and 34/171 (19.88%) in gynecology. Laparoscopy was done in 57/288 (19.79%) patients, 24/117 (20.51%) in pediatric surgery, and 19/171 (19.29%) in gynecology. The survival rate in benign masses was 100%, and 86.28% in malignancies.

**Conclusion:**

This study highlights the diverse spectrum of ovarian masses managed by pediatric surgeons and gynecologists. A laparoscopic approach combined with ovarian preservation, which was comparable between specialties, should be the preferred method for managing benign lesions whenever feasible. These findings underscore the need for standardized, collaborative guidelines between pediatric surgeons and gynecologists to ensure consistent and optimal management of ovarian masses in children.

## 1 Introduction

Ovarian lesions are the most common genital tract lesions in female children, accounting for 60%–70% of gynecologic lesions, and are primarily benign [1]. They are also the least common tumors in children, accounting for around 1% of all tumors with an incidence of 2.6 per 100 000 girls per year, and only 10% of these are malignant [1, 2, 3]. Diseases of the ovary are divided into two categories: non‐neoplastic and neoplastic lesions. Non‐neoplastic includes functional cysts, inflammatory and non‐inflammatory lesions. Neoplastic lesions include benign, borderline, and malignant tumors [1, 4, 5].

The incidence, clinical presentation, and histology of ovarian lesions in children differ from the adult population [6]. Children may present with non‐specific symptoms, ranging from abdominal pain to a distended abdomen. Some children may be asymptomatic, with an incidental finding of an abdominal mass [6]. Little is known about pediatric ovarian masses in developing countries, where late presentation, unavailability of facilities, and low index of suspicion lead to delayed diagnosis, advanced presentation, disease progression, and complications of the disease, leading to a reduced survival rate [7, 8].

Historically, the management of ovarian masses in children has been through surgical removal; however, the current advanced radiologic imaging and identification of tumor markers allow for more conservative management. Unlike in adults, where the management of ovarian tumors is oophorectomy, the management approach in children aims to preserve ovarian tissue to spare gonadal function and fertility, utilizing a minimally invasive surgery (MIS) approach [9, 10, 11].

In South Africa, Paediatric Surgery became a fully independent fellowship specialty in 2008, and the College of Paediatric Surgeons was established in 2010. However, as of 2018, there were only 2.6 pediatric surgeons per one million population. This limited number of pediatric surgeons relative to the disease burden has historically meant that gynecologists, who are more readily accessible in many areas, have treated pediatric patients with gynecological issues.

For many referring healthcare professionals, particularly in regions with fewer pediatric surgeons, gynecologists have often been the first point of contact for pediatric patients with gynecological concerns. As a result, these patients may be initially managed by gynecologists.

While the number of pediatric surgeons in South Africa has increased in recent years and referring clinicians are becoming more aware that pediatric surgeons, rather than gynecologists, should be the primary specialists for pediatric patients, there are still cases where patients are initially seen by gynecologists. These patients are subsequently referred to pediatric surgery for further management.

With little research and little known about pediatric ovarian lesions, their diagnosis, and management in South Africa and Africa as a whole, this study aimed to describe the profile and surgical management of ovarian masses in children 18 years and younger at two academic hospitals in Johannesburg from 1 January 2007 to 31 March 2022, in preparation to standardize surgical management at a national level. The hypothesis is that there is a variation in the management between the two sub/specialties due to differences in training and practice profile.

## 2 Materials and Methods

The study was a retrospective review of surgically biopsied or excised ovarian masses. It was conducted in the Pediatric Surgery and Gynecology Departments at Chris Hani Baragwanath Academic Hospital (CHBAH) and Charlotte Maxeke Johannesburg Academic Hospital (CMJAH). CHBAH is a 2680‐bed central academic hospital, and CMJAH is a 1200‐bed central academic hospital, both affiliated with the University of the Witwatersrand. The study was approved by the University of the Witwatersrand Human Research Ethics Committee (Medical) (M220536). It was a record review of children 18 years and younger who presented and were surgically managed for ovarian masses at CHBAH and CMJAH between 1 January 2007 and 31 March 2022. Patients were excluded if they had no surgical intervention, were treated but not operated on at the two study hospitals, if the specimens were pregnancy‐related specimens or involved disorders of sexual differentiation (DSD), and those cases with missing records.

The following data were collected regarding the patient profile: age, clinical presentation (presenting complaint, signs of precocious puberty and associated syndromes), investigations (radiological and tumor markers), histopathological diagnosis, and 5‐year survival outcome of malignant lesions. The surgical management data included: the treating surgical department, type of surgical intervention and approach, intraoperative staging, and chemotherapy.

The data were collected by one author (NM). Pathology reports were retrieved from the Central Data Warehouse (CDW) and the National Health Laboratory Service (NHLS) databases. The pathology reports were reviewed to compile a list of patients that were reviewed for inclusion in the study. Additional data were obtained from patient files, the pediatric surgical oncology database, and the hospitals' Picture Archiving and Communication System.

Data were analyzed using STATA version 14 (StataCorp. 2015. Stata Statistical Software: Release 14. College Station, TX: StataCorp LP). Age was described using means and standard deviations. The remainder of the data was described as frequencies and percentages where applicable.

## 3 Results

**FIGURE 1:**
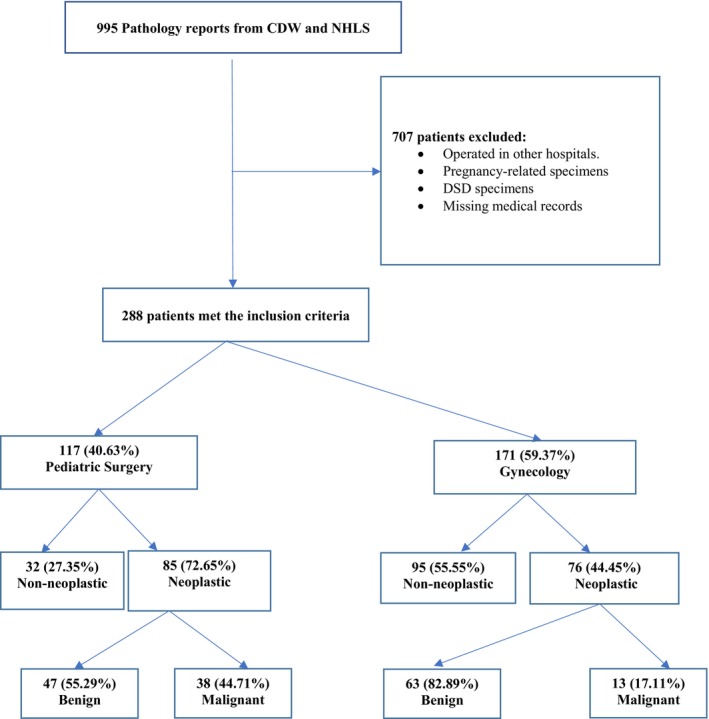
Sample realization. CDW, Central Data Warehouse; DSD, Disorders of Sexual Differentiation; NHLS, National Health Laboratory Service.

The sample realization is shown in Figure 1. Of the 288 patients, 117 (40.63%) presented to pediatric surgery and 171 (59.37%) to gynecology. The overall mean (SD) age was 13.34 years (±5.116), 12.53 (±5.115) in pediatric surgery, and 13.38 (±5.094) years in gynecology. The mean age (SD) for non‐neoplastic lesions was 13.35 (±5.122), 13.36 (±5.079) for neoplastic benign, and 13.35 (±5.072) for malignant disease. Fifty‐one (17.70%) had malignant disease; of the patients with malignant disease, 14 (27.45%) were 10 years and below, and 37 (72.54%) were 11 years and above. Table 1 shows a summary of the patient's clinical presentation. Two patients had Peutz‐Jeghers Syndrome, and four had polycystic ovarian syndrome (PCOS). Ten patients had associated precocious puberty.

**TABLE 1:** Clinical presentation.

Clinical presentation (*N* = 288)	Presenting symptom	*N*	%	Lesion	*N*	%
Main presenting complaint	Abdominal pain	149	51.73	Non‐ neoplastic	98	65.77
				Neoplastic benign	30	20.13
				Malignant	21	14.10
	Abdominal mass	135	46.88	Non‐ neoplastic	27	20
				Neoplastic benign	78	57.78
				Malignant	30	22.22
	Other	4	1.39	Non neoplastic	2	50
				Neoplastic benign	2	50
				Malignant	0	0
Associations *N* = 16	Precocious Puberty	10	3.47	Non‐neoplastic	0	0
				Neoplastic benign	0	0
				Malignant	10	100
	Peutz‐Jeghers syndrome	2	0.69	Non‐neoplastic	0	0
				Neoplastic benign	0	0
				Malignant	2	100
	Polycystic ovarian syndrome	4	1.39	Non‐neoplastic	3	75
				Neoplastic benign	1	25
				Malignant	0	0

Ultrasound imaging done in 212 (73.61%) patients, reported 199 (93.87%) to have unilateral disease and 13 (6.13%) bilateral disease. CT scan was done in 121 (42.01%) patients and only 11 (7.43%) of the 13 initially reported by sonar to have bilateral disease had bilateral disease. Tables 2 and 3 show radiological investigations and malignant tumors with associated tumor markers respectively. The surgical intervention, surgical approach, and histopathological diagnosis are shown in Tables 4 and 5.

**TABLE 2:** Radiological investigations.

Radiological investigation	Type of lesion	Cystic only		Solid only		Mixed	
		*N*	%	*N*	%	*N*	%
U/S *N* = 212	Non neoplastic	63	0.58	22	21.15	19	18.27
	Neoplastic benign	25	31.65	5	6.33	49	62.02
	Malignant	3	10.34	8	27.59	18	62.07
CT *N* = 121	Non neoplastic	10	37.04	9	33.33	8	29.63
	Neoplastic benign	14	23.73	3	5.08	42	71.19
	Malignant	3	8.57	8	22.86	24	68.57

**TABLE 3:** Malignant tumors and associated markers.

Type of malignant tumor *N* = 51	Total number of patients diagnosed	AFP	β‐HCG	CA 125
		Number of patients positive/tested	Number of patients positive/tested	Number of patients positive/tested
Germ cell tumors				
Yolk sac tumor	15	15/15 (100%)	5/11 (45.45%)	3/3 (100%)
Dysgerminoma	7	0/5 (0)	6/7 (85.71%)	3/4 (75%)
Choriocarcinoma	2	0/2 (0)	2/2 (100)	0
Immature teratoma	2	2/2 (100%)	0/2 (0)	0/2 (0)
Mixed germ cell tumor (Embryonal and yolk sac)	5	5/5 (100%)	3/5 (60%)	1/3 (75%)
Sex cord–stromal tumors				
Granulosa cell tumors	10	0/6 (0)	0/3 (0)	3/5 (60%)
Sex cord stromal tumor with annular tubules	2	2/2 (100%)	0/2 (0)	1/2 (50%)
Sertoli‐Leydig cell tumor	6	1/5 (20%)	1/5 (20%)	4/6 (66.67%)
Epithelial tumors				
Grade 3 mucinous epithelioid	1	0	0/1 (0)	1/1 (100%)
Other (Mesothelioma)	1	1/1 (100%)	1/1 (100%)	1/1 (100%)

**TABLE 4:** Surgical intervention and approach.

		Benign		Malignant	
		*N*	%	*N*	%
Open surgery *N* = 224 (77.78%)					
Pediatric surgery *N* = 91	Oophorectomy	41	77.36	38	100
	Ovarian sparing	12	22.64	0	
Gynecology *N* = 133	Oophorectomy	99	81.15	11	100
	Ovarian sparing	23	18.85	0	0
Minimal invasive surgery (MIS) *N* = 57 (19.79%)					
Pediatric surgery *N* = 24	Oophorectomy	14	58.33	0	0
	Ovarian sparing	10	41.67	0	0
Gynecology *N* = 33	Oophorectomy	20	64.52	2	100
	Ovarian sparing	11	35.48	0	0
Unspecified surgical approach *N* = 7 (2.43%)					
Pediatric surgery *N* = 2	Oophorectomy	2	100	0	0
	Ovarian sparing	0	0	0	0
Gynecology *N* = 5	Oophorectomy	5	100	0	0
	Ovarian sparing	0	0	0	0

**TABLE 5:** Histo‐pathological diagnosis.

Class of lesion *N* = 288	Type of lesion	Pediatric surgery		Gynecology	
Non‐neoplastic *N* = 127		*N* = 32	%	*N* = 95	%
	Follicular cyst of ovary	11	34.36	8	8.42
	Corpus luteum cyst	6	18.75	31	32.63
	Other unspecified ovarian cysts (simple cyst)^a^	8	25.01	25	26.32
	Pelvic inflammatory masses	3	9.38	14	14.74
	Torsion ovary	4	12.5	17	17.89
Neoplastic benign *N* = 110		*N* = 47	%	*N* = 63	%
	Surface epithelial tumors	7	14.89	20	31.75
	Sex cord–stromal tumors	4	8.51	3	4.76
	Germ cell tumors^b^	36	76.60	40	63.49
Neoplastic malignant *N* = 51	Surface epithelial tumors *N* = 1	*N* = 0	%	*N* = 1	%
	Grade 3 mucinous epithelioid	0	0	1	100
	Sex cord–stromal tumors *N* = 18	*N* = 9	%	*N* = 9	%
	Granulosa cell tumors (adult and juvenile)	4	44.45	6	66.67
	Sertoli‐Leydig stromal tumors^c^	3	33.33	3	33.33
	Sex cord stromal tumor with annular tubules^c^	2	22.22	0	0
	Germ cell tumors *N* = 31	*N* = 29	%	*N* = 2	%
	Yolk sac tumors	14	48.28	1	50
	Dysgerminomas	6	20.69	1	50
	Immature teratomas	2	6.90	0	0
	Mixed germ cell tumors	5	17.23	0	0
	Nongestational choriocarcinoma	2	6.90	0	0
	Miscellaneous tumors *N* = 1	*N* = 0	%	*N* = 1	%
	Mesothelioma	0	0	1	100

^a^ Three patients with unspecified cysts had polycystic ovarian syndrome.

^b^ One patient with mature cystic teratoma had polycystic ovarian syndrome.

^c^ Two patients with sex cord stromal tumors had Peutz‐Jeghers syndrome.

Of the 51 patients presenting with malignant tumors, pediatric surgery had 38/51 patients. A documented staging report was found in 18/38 patients. Pediatric Surgery used the Children Oncology Group (COG) staging system. Two patients were COG stage 1, 3 were stage 2, 9 stage 3, and 4 stage 4. Out of the 38 patients, 21 received chemotherapy of which 9 received a neo‐adjuvant carboplatin‐etoposide‐bleomycin regimen (JEB), 8 adjuvant therapy JEB, and 4 received adjuvant cisplatin‐etoposide‐bleomycin regimen (BEP).

In two cases, the decision for neo‐adjuvant chemotherapy was based on intraoperative laparoscopic biopsy of the ovarian masses, which were of unknown origin. These patients were initially taken to surgery with a suspected diagnosis of complicated appendicitis. During surgery, ruptured ovarian masses were discovered that were deemed inoperable at the time. Biopsies were then to aid in determining the appropriate course of treatment. For the remaining patients, the decision for neo‐adjuvant chemotherapy was guided by positive tumor markers and suggestive imaging findings that raised suspicion for malignancy.

Gynecology had 13/51 patients with malignant lesions, a documented staging report was found in 13/13 patients. Gynecology used the International Federation of Gynecology and Obstetrics (FIGO) staging system. Seven were FIGO stage 1, 4 stage 2, and 2 were stage 4. The 2 patients with stage 4 disease presented critically ill and demised without getting chemotherapy.

The 5‐year survival analysis showed no mortality in patients with non‐neoplastic and neoplastic benign lesions. Seven (13.72%) of the 51 patients with malignant lesions demised and hence the overall survival rate in the malignant cohort was 44/51 (86.28%). Five deaths were from Pediatric Surgery, and two were from Gynecology. In pediatric surgical patients, 1 patient with advanced yolk sac tumor died 10 days post‐surgery from overwhelming sepsis secondary to necrotic bowel post‐surgery, 2 deaths were from neutropenic sepsis post‐chemotherapy in patients with yolk sac tumors and 2 deaths were from patients diagnosed with advanced dysgerminoma and Sertoli Leydig cell tumor with lung metastasis. The 2 patients from Gynecology who demised had stage 4 yolk sac tumors and mesothelioma respectively.

## 4 Discussion

In this study, 288 cases were operated in 15 years, surpassing the expectations set by the authors and findings reported in other African literature and high‐income countries. For instance, in South‐Western Nigeria, only 24 cases were documented over 22.5 years [12], while Tunisia reported 98 cases in 15 years [13], and Australia recorded 244 cases over 19 years [14]. However, contrasting figures were observed in Shanghai, China, with 521 cases documented over 9 years [4], and in Texas, where 752 cases were reported over 10 years [15]. The variance between the findings of this study and those of the Shanghai study might be attributed to differences in population size, while Texas's higher case count could be linked to a more robust record‐keeping system. These disparities highlight the importance of considering regional factors and healthcare infrastructure when interpreting and comparing research findings.

Children diagnosed with ovarian lesions may seek treatment from either pediatric surgery or gynecology departments, leading to potentially differing management approaches. In the hospitals studied, the majority of patients 171/288 (59.37%) were seen by the gynecology department. This contrasts with findings from a study by Xac et al., where 68.3% of patients were seen by pediatric surgeons [15]. The variation in departmental presentations can be attributed to several factors. Before COVID‐19, the pediatric surgery department at CHBAH had an age limit of 10 years, which later increased to 14 due to bed shortages in adult wards. Additionally, it's possible that in Johannesburg, there's a prevailing assumption among clinicians that gynecologists exclusively manage ovarian pathology. In some cases, 4/288 (1.39%) patients presented to general surgical departments with symptoms suggestive of bowel obstruction or appendicitis, prompting intra‐operative involvement of gynecologists for management.

Non‐neoplastic and benign neoplastic lesions are more prevalent among pediatric patients than malignant ones, according to literature [1], This study also observed that non‐neoplastic and benign neoplastic lesions are the most common ovarian lesions in children. It further noted that the risk of malignancy rises with age, with the majority of patients diagnosed with malignancy being older than 10 years, consistent with findings from a Nigerian study [6].

Contrasting observations exist; in Tennessee, USA, a higher risk of malignancy was reported among younger patients [16], whereas Zhang et al. [4] found no variation in age distribution. In low‐to‐middle‐income countries, a low index of suspicion and delayed referrals may contribute to later presentations [7].

A review conducted by Heo et al. [6], concluded that clinical manifestations in children are generally nonspecific, but frequently include abdominal pain, abdominal distension, and the presence of a palpable mass. This aligns with our study's findings, where abdominal pain emerged as the most prevalent symptom 149/288 (51.73%). Patients with non‐neoplastic lesions predominantly presented with pain, while those with neoplastic benign and malignant lesions commonly exhibited an abdominal mass. Notably, Zhang et al. [4] reported that only 6% of patients with malignant lesions presented with a palpable mass. However, in our study, 135 out of 288 patients (46.89%) exhibited a palpable abdominal mass. This discrepancy may be attributed to the presence of more advanced disease in our study cohort compared to that reported by Zhang et al.

Precocious puberty and its related syndromes were uncommon in this study, consistent with findings from other research [14, 17]. Patients diagnosed with sex cord‐stromal tumors may exhibit precocious puberty, as evidenced by the 10 cases in our study, all of which involved sex cord‐stromal tumors (specifically granulosa cell tumors). Additionally, two patients (0.7%) diagnosed with Peutz‐Jeghers syndrome in our study presented with sex cord‐stromal tumors (one Leydig cell tumor and one sex cord‐stromal tumor with annular tubules), a linkage extensively documented in the literature [18, 19, 20]. Young et al. reported that 36% of patients with sex cord‐stromal tumors with annular tubules had Peutz‐jeghers syndrome [21]. In contrast, this study found that 50% of patients with sex cord‐stromal tumors with annular tubules had Peutz‐jeghers syndrome.

While some studies hypothesized a potential link between PCOS and ovarian cancer due to increased androgen exposure [22]; others found no association [23, 24]. Our study although not powered to do so did not find any correlation between PCOS and ovarian cancer; among the four patients with PCOS, three had unspecified simple cysts, and one had a cystic ovarian teratoma.

Ultrasound is utilized to characterize masses and assess for malignancy. Manegold‐Brauer et al. noted a low specificity and sensitivity of ultrasound in children [25]; however, How et al. found ultrasound to be moderately effective in detecting malignancy [14]. According to Heo et al. [6], ultrasound, CT, and MRI can aid in distinguishing between benign and malignant tumors, with radiological findings guiding treatment decisions. In our study, ultrasound was conducted on 212 and CT on 121 out of 288 patients (Table 2), the use and mode depending on the facilities and clinical presentation, results revealed that non‐neoplastic lesions were predominantly cystic (60%, 37% respectively), neoplastic benign lesions exhibited a mixed (cystic and solid) composition (62%, 71% respectively), and malignant lesions appeared solid (28%, 22% respectively) or mixed (62%, 69% respectively), some patients who did not undergo ultrasound, particularly those presenting with peritonitis, were taken directly to laparotomy or laparoscopy for diagnostic and therapeutic purposes. These findings align with existing literature [6] and correlated between Sonar and CT. Ultrasound detected bilateral disease in 13 patients, yet only 11 were confirmed on CT scans. Intraoperatively, only 5 of these cases were pathologically confirmed demonstrating shortcomings in our institutional diagnostic radiology, however, both ultrasound and CT scans facilitated diagnosis and informed potential treatment modalities for patients in our study.

Tumor markers play a vital role in screening, diagnosing, prognosticating, and monitoring treatment [26]. When combined with age, they can offer valuable diagnostic insights [27] and aid in providing a differential diagnosis [6]. However, in this study, not all patients underwent tumor marker testing due to the inclusion of emergency cases requiring immediate surgical exploration before further investigations. Nonetheless, for those tested, tumor markers assisted in formulating a differential diagnosis and guiding management decisions. Additionally, these markers confirmed that certain ovarian tumors can exhibit atypical hormonal activity, as evidenced by the 10 patients with granulosa cell tumors who presented with associated precocious puberty.

In this study, follicular cysts of the ovary were the most prevalent non‐neoplastic lesions among patients treated by pediatric surgeons, contrasting with corpus luteal cysts, simple cysts, and pelvic inflammatory masses more frequently observed in gynecology patients. Mature cystic teratoma emerged as the most common benign neoplastic lesion in both departments, aligning with findings from studies conducted in Nigeria [12], Tunisia [13], and Texas [15].

Germ cell tumors surpassed Sex cord and Surface epithelial tumors in prevalence across both departments, although older patients treated by gynecologists exhibited a higher incidence of Surface epithelial‐stromal tumors compared to those seen by pediatric surgeons. Yolk cell tumor and dysgerminoma were the most frequent malignant lesions among patients aged 10 and younger, differing from reports in other studies; Burkitt's lymphoma in Nigeria [12], juvenile granulosa cell tumors in Tunisia [13], and mixed germ cell tumors in Texas [15]. Sertoli‐Leydig cell tumors were predominantly observed in patients older than 10 years.

The preferred approach for patients with benign lesions and normal serum tumor markers, or when the diagnosis is uncertain, is laparoscopy [6]. This minimally invasive technique is known to reduce morbidity, postoperative complications, pain, hospital stay duration, and total hospital costs [11]. However, in our study, laparoscopy was performed on only 57 out of 288 patients (19.79%), with 24 out of 117 (20.51%) in pediatric surgery and 33 out of 171 (19.29%) in gynecology. The low utilization rate may be attributed to factors such as limitations in funding for laparoscopic equipment, the hierarchical structure of surgical care, and the expertise required for laparoscopic procedures, as noted by Choy et al. [28]. All patients who underwent MIS in pediatric surgery had benign diseases, while those with suspected malignant lesions underwent laparotomy. The type of open surgery performed varied depending on the clinical situation and the surgeon's assessment.

Most open surgeries were conducted via a standard midline incision or Pfannenstiel incision, depending on the location and size of the ovarian mass. The choice of approach was made based on the clinical presentation, the need for adequate exposure, and the surgeon's judgment regarding the safest and most effective method for the specific case.

Laparoscopy has been a challenging modality to adopt in our institutions for a variety of reasons. Key barriers include a lack of consumables, limited availability of laparoscopic equipment, and insufficient numbers of skilled surgeons proficient in laparoscopic techniques. Additionally, inconsistent electricity supply and difficulties in equipment maintenance have further hindered the widespread use of laparoscopy.

While these challenges have historically contributed to the low utilization of laparoscopy, we are pleased to report that improvements have been made in recent years. The availability of laparoscopic equipment has gradually increased, and more training opportunities have become available to surgeons, leading to greater expertise in this technique. As a result, we are seeing a slow but steady increase in the adoption of laparoscopy in clinical practice.

Two patients from gynecology, both with Juvenile granulosa and adult granulosa cell tumor FIGO 1A, underwent MIS as they required emergency explorations before further investigations. However, for malignant diseases, the gold standard is complete removal without rupture of the malignant lesion, preferably through laparotomy. Zhang et al. [4] reported that as many as 82% of their patients underwent laparoscopic surgery. Their study involved patients aged 9 to 19 years, while ours included patients aged 0 to 18 years. Additionally, their higher rate of pediatric laparoscopic surgeries suggests a more developed pediatric laparoscopic surgery program.

Eskander et al. [10] and Xac et al. [15] found that patients treated by gynecologists are more likely to undergo ovarian‐sparing surgery. In our study, ovarian‐sparing surgery was performed in 56 out of 288 patients (19.44%) with non‐neoplastic and benign neoplastic lesions across both disciplines, with 22 out of 117 (18.80%) in pediatric surgery and 34 out of 171 (19.88%) in gynecology. All patients with suspected malignancy preoperatively or intraoperatively from both departments underwent oophorectomies. Zhang et al. [4] conducted ovarian‐sparing surgery in 81.8% of cases with benign lesions and cautioned against ovarian cystectomy in malignant lesions due to the high recurrence rate.

Bilateral tumors of the ovary are relatively uncommon, with an estimated incidence ranging from 0.7% to 1.5%, and the majority are reported to be malignant [29]. They can occur synchronously or metachronous and may range from benign to malignant, presenting considerable challenges in management [30].

These tumors can exhibit the same or different histology, simultaneous occurrence of two tumors with different histology is uncommon [31]. In this study 1 patient had non‐gestational choriocarcinoma in the left ovary and mature cystic teratoma in the right ovary, 2 patients had bilateral benign ovarian teratomas, and 3 had bilateral atypical serous tumors.

Management of bilateral ovarian masses can be challenging, if malignancy is suspected, bilateral oophorectomy is acceptable [32]. The patient diagnosed with non‐gestational choriocarcinoma and mature cystic teratoma showed elevated B hCG levels and underwent laparotomy followed by bilateral oophorectomy. The two patients with bilateral mature cystic teratoma underwent bilateral oophorectomy, one via open surgery and the other through MIS. Majority (50%) of serous tumors are benign, 15% are borderline, while 35% are malignant [31]. The three patients diagnosed with atypical serous tumors had no documented tumor markers before surgery, and all underwent bilateral oophorectomy via open surgery.

The decision to perform a bilateral oophorectomy in pediatric patients is a complex and controversial issue, with no single approach capable of fully addressing all the factors involved. When bilateral ovarian malignancy is suspected, the surgical strategy should be based on oncologic principles, while also considering the balance between disease control and fertility preservation when appropriate. A comprehensive surgical staging procedure, which includes peritoneal washings, biopsies, omentectomy, and lymph node assessment, is essential to determine the extent of the disease. In cases where malignancy is confirmed, cytoreductive surgery may be necessary to reduce tumor burden. For young patients desiring fertility, unilateral oophorectomy with preservation of the contralateral ovary may be considered, particularly for germ cell tumors or low‐grade epithelial tumors. In certain cases, bilateral ovarian cystectomy, or ovarian‐sparing surgery, might be attempted if both ovaries are affected but functional tissue can be preserved. However, in cases of advanced or high‐risk malignancies, such as high‐grade epithelial ovarian cancer, bilateral salpingo‐oophorectomy (BSO) is typically required, and a total hysterectomy may be performed depending on the extent of the disease.

Many pediatric ovarian malignancies, such as germ cell tumors, are chemo sensitive, and chemotherapy regimens, such as BEP (bleomycin, etoposide, and cisplatin), may help shrink the tumor or enable ovarian preservation prior to surgery. Management of these cases should involve a multidisciplinary team, including pediatric surgeons, gynecologic oncologists, medical oncologists, and fertility specialists, to develop a treatment plan tailored to the tumor type, disease stage, and the patient's reproductive goals. This approach ensures the best possible balance between oncological safety and fertility preservation, individualized to meet the specific needs of each patient.

Two patients from the gynecology department were diagnosed with stage 4 pulmonary disease at 18 years old and passed away during the same admission. One patient was HIV positive but not yet undergoing treatment and was diagnosed with advanced mesothelioma. The other patient was diagnosed with a yolk sac tumor.

Among the five patients from pediatric surgery, all passed away within 2 years of diagnosis. The youngest was 18 months old and had a yolk cell tumor, while the oldest was 13 years old and had a stage 4 Sertoli Leydig cell tumor with lung metastasis.

A limitation of this study is its contextual nature, as it was conducted exclusively in two academic hospitals in Johannesburg. This may limit the generalizability of the results to other hospitals. We acknowledge that the involvement of both paediatric surgeons and gynaecologists in the management of the patients could introduce potential intervention bias, as the treatment protocols and approaches may differ between these two specialties. While both groups are well‐trained in the management of ovarian masses, there may be variations in how they approach diagnosis, treatment, and follow‐up care, which could influence outcomes. We take this into account and recognize that this represents a limitation of the study.

We also note that the involvement of multiple specialties reflects the real‐world clinical setting in South Africa, where paediatric surgery and gynaecology are closely intertwined due to limited paediatric surgical resources in our region. However, we agree that this variability in care could impact the generalizability of our findings, particularly with regard to intervention choices and management strategies.

To address this limitation, we plan to emphasize the importance of standardizing care pathways and improving multidisciplinary collaboration in future research, which may help mitigate intervention bias moving forward.

## 5 Conclusion

This study highlights the diverse spectrum of ovarian masses managed by pediatric surgeons and gynecologists, with most being benign. Malignant lesions were more common in patients aged 10 and above, emphasizing the need for thorough preoperative evaluation using tumor markers and imaging to guide surgical decision‐making.

Minimally invasive and ovarian‐sparing surgery, which was comparable between specialties, is crucial for optimizing outcomes. A laparoscopic approach combined with ovarian preservation should be the preferred method for managing benign lesions whenever feasible.

These findings underscore the need for standardized, collaborative guidelines between pediatric surgeons and gynecologists to ensure consistent and optimal management of ovarian masses in children.

## Author Contributions

**Nkhensani C. Mashaba:** conceptualization (lead), data curation (lead), formal analysis (lead), investigation (lead), methodology (lead), project administration (lead), visualization (lead), writing – original draft (lead), writing – review and editing (lead). **Langanani Mbodi:** conceptualization (equal), data curation (equal), formal analysis (lead), investigation (lead), methodology (equal), project administration (lead), resources (equal), supervision (lead), validation (lead), visualization (equal), writing – original draft (equal), writing – review and editing (lead). **Ellen M. Mapunda:** conceptualization (equal), data curation (supporting), formal analysis (supporting), investigation (supporting), methodology (supporting), project administration (supporting), supervision (supporting), validation (supporting), visualization (supporting), writing – original draft (supporting), writing – review and editing (supporting). **Tanvier Omar:** conceptualization (equal), data curation (supporting), formal analysis (supporting), investigation (supporting), methodology (supporting), project administration (supporting), resources (supporting), supervision (supporting), validation (supporting), visualization (supporting), writing – original draft (supporting), writing – review and editing (equal). **Derek S. Harrison:** conceptualization (equal), data curation (supporting), formal analysis (equal), investigation (supporting), methodology (supporting), project administration (supporting), supervision (lead), validation (equal), visualization (equal), writing – original draft (supporting), writing – review and editing (equal).

## Conflicts of Interest

The authors declare no conflicts of interest.

## Data Availability

The data repository utilized (such as cancer data from Johannesburg) and specific data (regarding ovarian tumors in children) is available upon request from the primary authors and gatekeeper (the National Health Laboratory Services) in South Africa.
